# Fontan-Associated Liver Disease (FALD) in the EUROFontan Experience. An Insight into European Awareness

**DOI:** 10.1007/s00246-025-03781-0

**Published:** 2025-03-13

**Authors:** Anna Gozzi, Luca Vedovelli, Emma Bergonzoni, Irene Cao, Emanuela Angeli, Rossana Zanoni, Roberta Biffanti, Gianfranco Butera, Kostantinos Dimopoulos, Alvaro Gonzalez Rocafort, Mark G. Hazekamp, Andrzej Kansky, Marien Lenoir, Thomas Martens, Giovanni Meliota, Bart Meyns, Matej Nosal, Carlo Pace Napoleone, Friso M. Rijnberg, Eva Sames Dolzer, Giuseppe Scrascia, Ugo Vairo, Joeri Van Puyvelde, Giovanni Di Salvo, Claudia Montanaro, Massimo A. Padalino

**Affiliations:** 1 Pediatrics Unit, San Bortolo Hospital, Vicenza, Italy; 2 Unit of Biostatistics, Epidemiology and Public Health, Department of Cardiac, Thoracic and Vascular Sciences and Public Health, University of Padova, Padua, Italy; 3 Pediatric and Congenital Cardiac Surgery, Department of Cardiac, Thoracic and Vascular Sciences and Public Health, University of Padova, Padua, Italy; 4 Pediatric Cardiac Surgery and Adult Congenital Heart Disease Program, Department of CardioThoracic and Vascular Medicine, IRCCS, Azienda Ospedaliero-Universitaria Di Bologna, Bologna, Italy; 5 Pediatric Cardiology Unit, Department of Woman and Child’s Health, University of Padova, Padua, Italy; 6 Department of Cardiac Surgery, Cardiology and Heart Lung Transplant, Bambino Gesù Children’s Hospital IRCCS, Rome, Italy; 7 Adult Congenital Heart Centre and Centre for Pulmonary Hypertension, Royal Brompton Hospital, Royal Brompton & Harefield Hospitals, Guy’s and St Thomas’ NHS Foundation Trust, London, UK National Heart and Lung Institute, Imperial College London, London, UK; 8 Servicio de Cirugía Cardiaca Infantil y Cardiopatías Congénitas del Adulto, Alianza Estratégica Hospital Universitario La Paz-Hospital Universitario Ramón y Cajal, Madrid, Spain; 9 Leiden University Medical Center (LUMC), Leiden, Netherlands; 10 Congenital Cardiac Surgery, Medical University of Warsaw, Warsaw, Poland; 11 Department of Congenital Heart Surgery, La Timone Children Hospital, Marseille, France; 12 Department of Cardiac Surgery, University Hospital of Ghent, Ghent, Belgium; 13 Department of Pediatric Sciences, Pediatric Cardiology, Giovanni XXIII Pediatric Hospital, Bari, Italy; 14 Department of Cardiac Surgery, University Hospitals Leuven, Louvain, Belgium; 15 National Institute of Cardio-Vascular Diseases, Childrens Heart Center, Bratislava, Slovakia; 16 Pediatric Cardiac Surgery Department, Regina Margherita Children’s Hospital, Turin, Italy; 17 Division of Pediatric and Congenital Heart Surgery, Kepler University Hospital, Linz, Austria; 18 Department of Pediatric Sciences, Pediatric Cardiac Surgery Unit, Giovanni XXIII Pediatric Hospital, Bari, Italy; 19 Pediatric and Congenital Cardiac Surgery, Department of Precision and Regenerative Medicine and Jonian Area, University of Bari Aldo Moro, Bari, Italy

**Keywords:** FALD, Fontan-associated liver disease, Congestive hepatopathy, Fontan, Follow-up

## Abstract

Fontan-Associated Liver Disease (FALD) is a dramatically emerging problem even if not precisely defined in term of debated diagnosis and surveillance protocols. We analyze FALD prevalence, clinical impact and implications in a European cohort of patients. It’s a retrospective observational multicenter study including Fontan patients operated between 1990 and 2022. Anatomical, clinical, surgical and liver-related data were collected, defining FALD as a spectrum of time-related structural–functional liver modifications due to congestive hepatopathy (from mild liver fibrosis to liver cirrhosis and hepatocellular carcinoma) diagnosed through multiparametric evaluations. 14 centers routinely conducted liver assessment after Fontan completion. Out of 2141 patients, 343 (16%) were diagnosed with FALD (M/F = 198/145; median age 18 years, IQR 15–26) with a median follow-up time of 14 years (IQR 9–20) from Fontan surgery. Among these, there were 19 (5.5%) deaths, 5 (26.3%) of whom related to advanced liver disease/cancer. FALD showed no significant association with gender (*p* = 0.4, adjusted *p*-value = 0.5), dominant ventricular morphology (*p* = 0.060, adjusted *p*-value = 0.086) nor surgery type (*p* = 0.3, adjusted *p*-value = 0.4). Significant association emerged between FALD and fenestration absence (*p* < 0.001, adjusted *p*-value < 0.001), systemic ventricular (*p* < 0.001, adjusted *p*-value < 0.001) and atrio-ventricular valve (*p* < 0.001) dysfunction, III-IV NYHA classes (*p* < 0.001, adjusted *p*-value < 0.001), tachyarrhythmias (*p* < 0.001) and liver stiffness ≥ 22 kPa on transient elastography (*p* < 0.001, adjusted *p*-value < 0.001). The analysis demonstrated no significant association between FALD and abnormal liver function tests (*p* = 0.2), heart transplantation (*p* = 0.6, adjusted *p*-value = 0.6), worse survival (*p* = 0.38). This study shows significant mortality related to FALD, which is also associated to clinical signs of failing Fontan circulation, stressing the pressing need of universally shared diagnostic criteria and surveillance protocols, to prevent and/or early-identify FALD and its more lethal complications.

## Introduction

Since 1970, the Fontan operation has allowed survival of hundreds of patients with single ventricle type congenital heart diseases. The Fontan principle implies the connection of the systemic venous return to the pulmonary arterial circulation (the cavopulmonary concept), separating systemic and pulmonary vascular beds, preventing a complete mixing in the single ventricle and mimicking a pseudophysiological circulation [1, 2]. Currently this procedure has substantially elevated survival rates of patients with single heart physiology: ongoing advancements in both surgical techniques and post-surgical management have contributed to significant improvements in both early and long-term survival. Consequently, individuals undergoing the Fontan operation and contributing to the so called “Fontan epidemic” are now reaching adulthood, with reported 20- and 30-year survival rates of 61 and 43%, respectively [3–8]. Notably, patients who completed a Fontan palliation after 2001 have reported even better outcomes, achieving a 10-year survival rate of 95% [6].

Given the increased survival, the long-term complications in this cohort open a new up-coming scenario. Among these, the so called FALD (Fontan-Associated Liver Disease) includes a wide spectrum of time-related structural and functional liver modifications that flourish through several stages. A well-established risk factor is time since surgery (especially more than 10 years) [9], together with the following suggested multiple factors: [1, 10–17]

- Risk factors related to surgery: atriopulmonary technique versus the total cavo-pulmonary connection concept, lateral tunnel versus extracardiac conduit, Fontan conduit stenosis/thrombosis;
- Risk factors related to Fontan circuit hemodynamic: reduced preload to the systemic ventricle for body surface area and cardiac output (with subsequent chronic arterial ischemia), progressive systolic-diastolic ventricular and atrioventricular valve dysfunction, increased pulmonary capillary pressure, increased splanchnic venous congestion and reduced lymphatic return through the thoracic duct;
- Risk factors related to cardiovascular events: cardiac arrhythmias and thrombosis;
- Other risk factors: pulmonary atresia as main diagnosis, hepatotropic virus infection, drug-induced hepatotoxicity (e.g. amiodarone).

FALD includes a broad variety of hepatic functional changes (laboratory evidences of hepatic injury or impaired synthesis) and structural modifications induced by hepatic altered passive flow and venous congestion, due to chronically elevated central venous pressures, low cardiac preload and output, lack of pulsatile flow and lymphatic overflow with obstruction [18]. The driving force for these alterations is passive chronic liver congestion due to the absence of a functional subpulmonic ventricle, but even before (even in utero) patients with a functional single ventricle undergo hypoxia and cardiovascular compromise that contribute to liver injury [19, 20]. Therefore some degree of liver anomalies due to congestive hepatopathy is found almost universally in Fontan patients. FALD includes a wide spectrum of manifestations [21, 22]: asymptomatic initial liver congestion and sinusoidal dilation [23], fibrosis without portal hypertension (with possible regenerative nodules) [24], advanced fibrosis with compensated/decompensated cirrhosis and portal hypertension (with eventual focal nodular hyperplasia, hepatocellular carcinoma, ascites, bleeding from esophagogastric varices or hepatic encephalopathy) [25–32]. The first evidence of cardiac cirrhosis after Fontan palliation was described in 1983 [33].

However, currently FALD lacks specific diagnostic and staging criteria and its definition is multiparametric. According to the current literature, it may reasonably include multidisciplinary data and multimodality imaging, that can be gradually implemented as FALD flourishes. The diagnostic-surveillance flow is driven by the combined interpretation of clinical informations, serologic data, radiological (cardiac and liver) findings, along with histological evidences when available, supported by predictive scores related to advanced FALD [10, 34–44].

Exact nature, frequency, severity and reversibility of chronic hepatic dysfunction in this setting are largely unknown. Just little literature exists about evidences on FALD diagnostic definition, surveillance protocols and prognostic impact. All the existing studies are limited by sample size and lack of universal consensus in the diagnostic-surveillance flow [45]. As not all patients develop liver-related complications, non-invasive and eventually invasive methods are needed to make early diagnosis, identifying patients at risk requiring close monitoring and preventive/curative treatments. The ideal methods for surveillance of Fontan hemodynamic status and associated liver damage (especially after 10 years after Fontan completion [18, 46, 47]) are still under investigation.

The aim of this multicenter European study is to analyze the current status of diagnosis, prevalence, and implications of Fontan-associated liver disease (FALD) during long-term follow-up after Fontan completion in a large cohort of European patients from the EUROFontan registry. This study seeks to highlight FALD as an emerging issue within the fields of congenital cardiology, cardiac surgery, and gastroenterology, despite lack of worldwide shared specific FALD diagnostic criteria. The number of Fontan patients potentially affected by FALD is dramatically increasing, yet the condition remains poorly defined in terms of diagnosis and surveillance protocols.

## Materials and methods

Study design: This is a retrospective observational multicenter clinical study known as EUROFontan, involving patients who underwent Fontan completion between 1990 and 2022. The study encompasses 21 major congenital heart centers across Europe. Data collection took place through a shared REDCAP database between December 2021 and January 2023, covering various parameters such as clinical, surgical, laboratory and instrumental data. The focus of this analysis is on patients diagnosed with Fontan-Associated Liver Disease (FALD) during the long-term follow-up.

Fontan-Associated Liver Disease: In accordance with the multiparametric surveillance evaluation outlined in international literature, FALD was defined as a spectrum of time-related structural and functional liver modifications attributed to congestive hepatopathy. This spectrum ranges from mild liver fibrosis to liver cirrhosis and hepatocellular carcinoma. The centers included in this study were asked to indicate whether a clinical diagnosis of Fontan-associated liver disease (FALD) was present (answering "yes" or "no") based on clinical, laboratory, and instrumental data, as suggested by Emamaullee et al. (36). However, they were not required to specify the diagnostic evidence used to make the FALD diagnosis for each patient. Specific data supporting the diagnosis were not requested in the original database collection, given the considerable variability that may arise due to the absence of a universal diagnostic approach. The long-term follow-up included the following data, when available: date of birth, gender, primary cardiac diagnosis, dominant ventricular morphology, presence of a secondary additional ventricular chamber, occurrence of heterotaxy syndrome, persistence of fenestration, surgical details of Fontan completion, age at follow-up, length of follow-up, any late deaths during follow-up along with their causes, functional classification according to the New York Heart Association (NYHA), laboratory parameters (ALT, AST, bilirubin, GGT, protein, hepatotropic viral/non-alcoholic markers) and instrumental data (such as evidence of sinus rhythm versus new onset of arrhythmia complications, echocardiographic qualitative assessment of systemic ventricular function and AV valve function, liver and spleen stiffness, inferior vena cava diameter, spleen enlargement at abdominal US and its diameter).

Inclusion criteria: all patients who underwent a Fontan completion between 1990 and 2022, and who were diagnosed with FALD at late term follow-up.

Statistical analysis: Descriptive statistics were summarized using frequencies and percentages (n, %) for categorical variables, and medians with interquartile ranges (IQR) for continuous variables. Inferential statistics involved Pearson’s Chi-squared test and Fisher’s Exact Test, to assess the association between categorical variables. The Wilcoxon rank sum test was utilized for comparing medians of continuous variables. Additionally, Fisher’s exact test was applied where appropriate. To account for the risk of Type I error due to multiple comparisons, a false discovery rate (FDR) correction was employed. Survival between FALD and non-FALD patients was investigated using Kaplan-Meyer curves and log-rank test.

## Results

Among the 21 centers participating in the EUROFontan study, only 14 routinely assessed liver function during long-term follow-up after Fontan completion. Of the 3,499 patients included in the EUROFontan database between December 2021 and January 2023, only 2,141 underwent liver-related assessments. Among these patients, FALD was diagnosed in 343 cases (16%), with a median age at follow-up of 18 years (IQR 15–26). The median follow-up time from Fontan surgery for patients with FALD was approximately 14 years (IQR 9–20) (see Table 3 for follow-up characteristics of EUROFontan patients included in this study). This analysis revealed that FALD patients were protagonists of a longer follow-up than non-FALD ones (more than 10 years in 70% of FALD cases, versus 53% in non-FALD Fontan patients).

In this series, 198 (58%) FALD patients were males, with no significant association with gender (*p* = 0.4, adjusted *p*-value = 0.5).

The most prevalent anatomical diagnosis was tricuspid atresia and pulmonary atresia with intact ventricular septum (30% of patients), with no significant association with FALD (*p* = 0.004, adjusted *p*-value = 0.007).

A functional single right or left ventricle was present in 41% and 56% of them, respectively. A secondary ventricular chamber was described in 31% of FALD patients, with heterotaxy syndromes in 8.2% of cases. This study failed to show any significant association between FALD and dominant ventricular morphology (*p* = 0.060, adjusted *p*-value = 0.086), a secondary additional ventricular chamber (*p* = 0.3, adjusted *p*-value = 0.4), and heterotaxy syndromes (*p* = 0.3, adjusted *p*-value = 0.4).

Furthermore, no significant association was found between FALD and the type of Fontan operation (*p* = 0.3, adjusted *p*-value = 0.4). However, the absence of fenestration during Fontan surgery was significantly associated with the diagnosis of FALD (*p* < 0.001, adjusted *p*-value < 0.001). All the demographic, surgical and early management characteristics of EUROFontan patients are described in detail in Tables 1, 2.

**Table 1:** Demographic features of EUROFontan patients included in this study

Characteristics	N	No FALD, N = 1,798^*1*^	FALD, N = 343^*1*^	p-value^*2*^	adjusted p-value^*3*^
Gender	2,136			0.4	0.5
Female		717 (40%)	145 (42%)		
Male		1,076 (60%)	198 (58%)		
Unknown		5	0		
Fundamental cardiac diagnosis	2,141			0.004	0.007
Double inlet left ventricle, Double inlet right ventricle		289 (16%)	58 (17%)		
Hypoplastic Left Heart Syndrome (HLHS), Hypoplastic left heart complex		437 (24%)	54 (16%)		
L-Transposition of the Great Arteries, D-Transposition of the great Arteries, Double outlet right ventricle, Double outlet left ventricle, Mitral atresia, Criss cross heart, Ebstein's Anomaly		407 (23%)	90 (26%)		
Tricuspid Atresia, Pulmonary atresia with intact ventricular septum		534 (30%)	104 (30%)		
Unbalanced Complete Atrioventricular Canal Defect (CAVC)-left dominant, Unbalanced Complete Atrioventricular Canal Defect (CAVC)-right dominant		131 (7.3%)	37 (11%)		
Dominant ventricular morphology	2,140			0.060	0.086
Left ventricle		915 (51%)	191 (56%)		
Right ventricle		783 (44%)	141 (41%)		
Other		100 (5.6%)	10 (2.9%)		
Unknown		0	1		
Secondary additional ventricular chamber presence	2,126	499 (28%)	104 (31%)	0.3	0.4
Unknown		12	3		
Heterotaxy syndrome	2,141			0.3	0.4
No		1,682 (94%)	314 (92%)		
Unknown/not documented		8 (0.4%)	1 (0.3%)		
Yes- subset not known; yes- asplenia syndrome (subset with components of bilateral right sideness, usually associated with absence of spleen, right isomerism); yes, polysplenia syndrome (subset with components of bilateral left sideness, usually associated with multiple spleens, left isomerism)		108 (6.0%)	28 (8.2%)

**Table 2:** Surgical and early management characteristics of EUROFontan patients included in this study

Characteristics	N	No FALD, N = 1,798^*1*^	FALD, N = 343^*1*^	p-value^*2*^	Adjusted p-value^*3*^
Type of Fontan operation	2,139			0.3	0.4
Atrial-pulmonary connection (Kreutzer), Atrial-ventricular connection (Bjork)		21 (1.2%)	8 (2.3%)		
Hepatic vein to Azygous Baffle		17 (0.9%)	2 (0.6%)		
TCPC,Extracardiac conduit,Fenestrated,or Non-Fenestrated		1,591 (89%)	304 (89%)		
TCPC,Lateral Tunnel,Fenestrated or Non-Fenestrated		168 (9.3%)	28 (8.2%)		
Unknown, not documented		1	1		
Presence of fenestration	2,139	1,352 (75%)	178 (52%)	< 0.001	< 0.001
Unknown		1	1		
Antiaggregation therapy at discharge	1,104	395 (42%)	87 (50%)	0.056	0.085
Unknown		867	170		
Pulmonary vasodilators at discharge	1,033	97 (11%)	22 (18%)	0.023	0.039
Unknown		890	218		
Antyarrhythmic medications at discharge	954	47 (5.5%)	21 (22%)	< 0.001	< 0.001
Unknown		939	248		

FALD patients presented significant atrio-ventricular valve dysfunction (*p* < 0.001), worse functional capacity (18.5% in NYHA ≥ class III versus 5% of non-FALD patients, *p* < 0.001, adjusted p-value < 0.001) and systemic ventricular dysfunction (*p* < 0.001, adjusted *p*-value < 0.001). In addition, we found a significant association between FALD and supraventricular tachyarrhythmias (*p* < 0.001) during the long-term follow-up (Table 3).

**Table 3:** Follow-up characteristics of EUROFontan patients included in this study

Characteristics	N	No FALD, N = 1,798^*1*^	FALD, N = 343^*1*^	p-value^*2*^	adjusted p -value^*3*^
Median age at follow-up	2,141	15 (10–20)	18 (15–26)	< 0.001	< 0.001
Follow-up time since Fontan completion (length of follow-up)	2,141	11 (5–16)	14 (9–20)	< 0.001	< 0.001
Follow-up more than 10 years since Fontan	2,141	953 (53%)	241 (70%)	< 0.001	< 0.001
Cause of late death (advanced liver disease/cancer)	2,141	0 (0%)	5 (1.5%)	< 0.001	< 0.001
Late surgical adverse event (heart transplantation)	2,128	33 (1.8%)	5 (1.5%)	0.6	0.6
Systemic ventricular function	2,118			< 0.001	< 0.001
Normal		1,398 (79%)	193 (57%)		
Mild dysfunction		227 (13%)	61 (18%)		
Mild to moderate dysfunction		58 (3.3%)	16 (4.7%)		
Moderate dysfunction		37 (2.1%)	8 (2.3%)		
Moderate to severe dysfunction		13 (0.7%)	4 (1.2%)		
Severe dysfunction		13 (0.7%)	5 (1.5%)		
Not assessed		18 (1.0%)	52 (15%)		
Unable to assess		13 (0.7%)	2 (0.6%)		
Unknown		21	2		
Systemic atrio-ventricular valve function (multiple choice questions)	1,725	1,533	192	< 0.001	
Normal		652(43%)	42(22%)		
Prosthetic valve with normal function		2(0.1%)	0(0%)		
Mild regurgitation		673(44%)	103(54%)		
Moderate regurgitation		185(12%)	39(20%)		
Severe regurgitation		19(1.2%)	8(4.2%)		
Sinus rhythm	2,109	1,498 (85%)	278 (82%)	0.14	0.2
Unknown		30	2		
Type of arrhythmia (multiple choice questions)	1,486			< 0.001	
AV block		23 (1.7%)	2 (1.4%)		
Sinus node dysfunction		38 (2.8%)	9 (6.3%)		
Pacemaker induced rhythm		44 (3.3%)	10 (7.0%)		
Atrial/Supraventricular Tachyarrhythmia (atrial fibrillation/futter/tachycardia)		70 (5.2%)	19 (13.0%)		
Re-entrant supraventricular tachyarrhythmias		13 (1.0%)	0 (0%)		
Ectopic atrial rhythm		73 (5.4%)	11 (7.7%)		
Junctional rhythm		106 (7.9%)	18 (13%)		
Non sustained ventricular tachyarrhythmias (3 beats or more with spontaneous resolution)		6 (0.4%)	2 (1.4%)		
Ventricular tachyarrhythmia (Ventricular Tachycardia, Ventricular fibrillation)		8 (0.6%)	1 (0.7%)		
Other		3 (0.2%)	2 (1.4%)		
None		950 (74%)	67 (47%)		
NYHA status	2,110			< 0.001	< 0.001
Class I		1,101 (62%)	147 (43%)		
Class II		559 (32%)	131 (38%)		
Class III		86 (4.9%)	51 (15%)		
Class IV		23 (1.3%)	12 (3.5%)		
Unknown		29	2		
NYHA Class III-IV	2,110	109 (6.2%)	63 (18%)	< 0.001	< 0.001
Antithrombotic therapy during follow-up					
Anticoagulation with vit. K antagonists (Warfarin)		355 (20%)	85 (25%)		
Anticoagulation with Other Anticoagulants Agents		67 (3.8%)	12 (3.5%)		
Anti-aggregation only		1,241 (71%)	221 (64%)		
Combined anticoagulation/antiaggregation		14 (0.8%)	5 (1.5%)		
None		78 (4.4%)	20 (5.8%)		
Unknown		43	0		
Pulmonary vasodilator therapy during follow-up					
no		1,222 (92%)	187 (72%)		
ET inhibitors (Bosentan and similar)		26 (2%)	23 (8.9%)		
PDE inhibitors (Sildenafil and similar)		77 (5.8%)	41 (16%)		
Combined therapy		7 (0.5%)	7 (2.7%)		
Other		0 (0%)	1 (0.4%)		
Unknown		466	84		

Among the collected liver/spleen data (Table 4), diagnosis of FALD during the long-term follow-up was significantly associated with *Fibroscan*-derived liver stiffness ≥ 22 kPa (*p* < 0.001, adjusted p-value < 0.001), but it was not associated to abnormal blood liver function test (*p* = 0.2).

**Table 4:** Liver and spleen data of EUROFontan patients included in this study

**Characteristics**	**N**	**No FALD**, N = 1,798^*1*^	**FALD**, N = 343^*1*^	**p-value**^*2*^	**adjusted p-value**^*3*^
**Fibroscan liver stiffness value (kPa)**	256	14 (10–18)	18 (13–24)	< 0.001	< 0.001
Unknown		1,698	187		
**Liver function test abnormalities (multiple choice questions)**	434	272	162	0.2	
ALT		42 (15%)	18 (11%)		
AST		31 (11%)	14 (8.6%)		
Bilirubin		72 (26%)	43 (27%)		
GGT		116 (43%)	74 (46%)		
Other		1 (0.4%)	2 (1.2%)		
Protein level lower than normal		10 (3.7%)	7 (4.3%)		
Viral hepatitis/ Non Alcoholic injury		0 (0%)	2 (1.2%)		
**Inferior vena cava diameter (mm)**		17.0 (14.8–19.0)	17.5 (13.5–19.8)		
Unknown		1,699	321		
**Abnormal alpha 1-antitrypsin concentration in stool (greater than normal range of normality)**		5 (5.0%)	5 (19%)		
Unknown		1,698	316		
**Spleen enlargement at abdominal US**		49 (30%)	46 (34%)		
Unknown		1,633	207		
**Spleen greater diameter (cm)**		11.5 (10.00–13.00)	12.00 (10.45- 13.00)		
Unknown		1,721	284		
**Spleen stiffness-fibroscan value (Kpa)**		26 (20–30)	33 (23–44)		
Unknown		1,754	319		

**Fig. 1:**
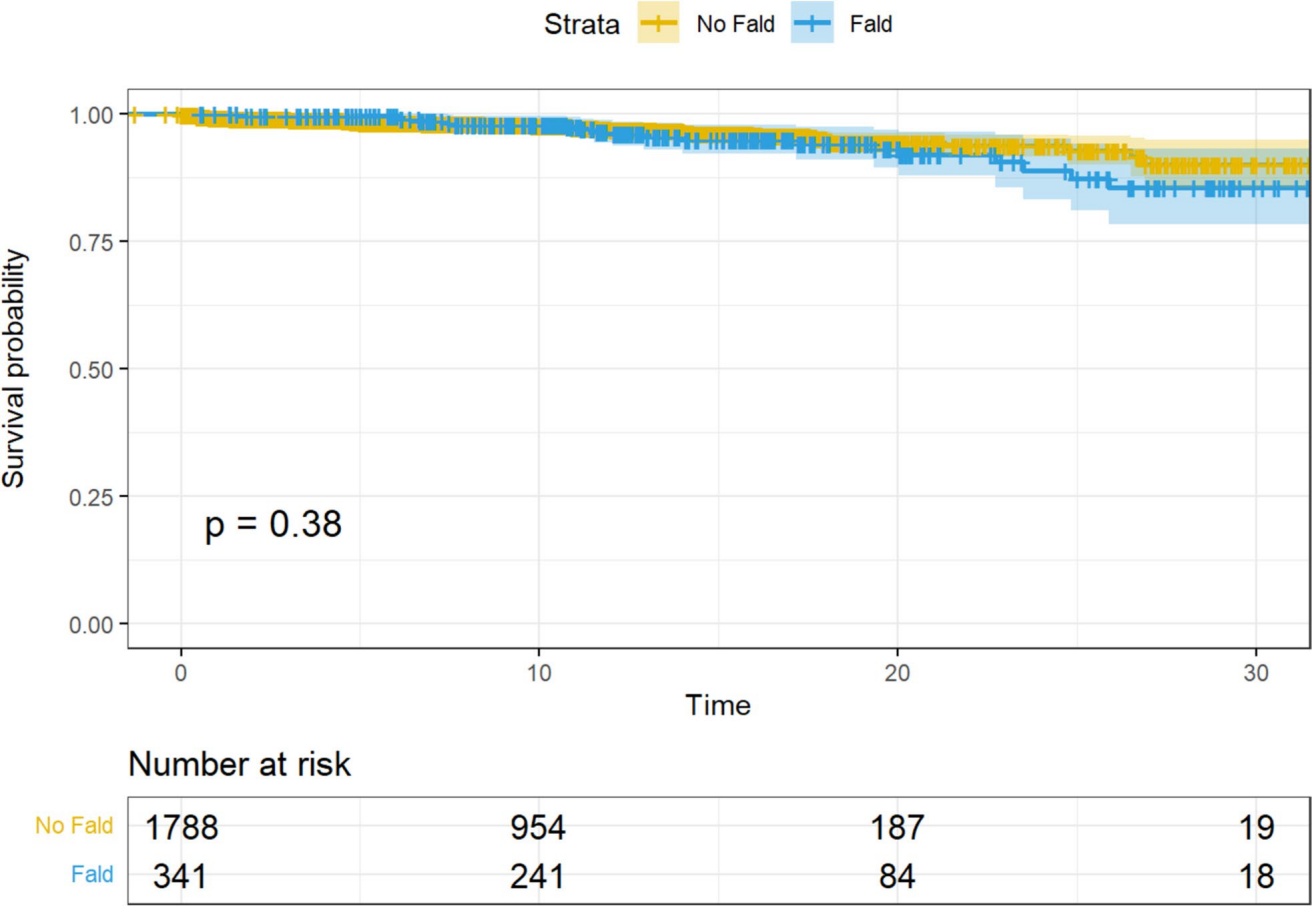
Decrease in survival probability after Fontan completion during the long-term follow-up, with no evidence of a significant association with worse survival in FALD patients

During the long-term follow-up, there were 24 deaths among non-FALD patients (1.3%) versus 19 (5.6%) in the FALD group. Among the latter, 5 patients died for advanced liver disease/cancer (accounting for 26% of deaths in FALD patients). Last, we observe neither a significant difference in rate of heart transplant (1.8% of non-FALD and 1.5% of FALD patients, *p* = 0.6, adjusted *p*-value = 0.6) nor in worse survival (*p* = 0.38, Fig. 1) in FALD patients.

## Discussion

Fontan-associated liver disease (FALD) encompasses a wide range of time-related structural and functional liver changes resulting from the chronic passive liver congestion that inevitably follows the Fontan procedure. These changes range from mild liver fibrosis to cirrhosis and hepatocellular carcinoma (36). Although the mechanisms underlying FALD are multifactorial and not yet fully understood, FALD significantly impacts the lives of Fontan patients by increasing the risk of liver dysfunction-related complications, such as hepatocellular carcinoma, gastroesophageal variceal bleeding, ascites, spontaneous peritonitis, hepatorenal syndrome, and encephalopathy. It also contributes to Fontan failure, which can present as protein-losing enteropathy, arrhythmias, pulmonary hypertension, plastic bronchitis, and reduced cardiac output. [48].

This preliminary report, focusing on FALD, demonstrates an overall prevalence of 16% in our multicenter cohort, highlighting European still inadequate awareness and emphasizing the urgent need for routine multidisciplinary and multimodality imaging work-ups to prevent the lethal outcomes observed in this cohort. Literature indicates a rising prevalence of FALD post-Fontan surgery, particularly after 10 years after Fontan completion, although FALD can occur even without Fontan failure. This underscores the necessity for early surveillance diagnostic protocols, as we infer that the cumulative risk of developing severe FALD begins shortly after Fontan completion and increases over time, reaching over 10% and more than 40–50% at 10 and 35 years, respectively [18, 49]. FALD represents a distinct clinical entity and challenge, evolving through various stages and impacting overall health and systemic organ function [21]. Currently, the optimal imaging modalities and follow-up strategies for liver surveillance remain subjects of ongoing debate.

The increased risk of liver dysfunction-related complications and Fontan failure negatively impacts life expectancy [48], as Fontan physiology influences the intricate interplay between the Fontan circuit and extracardiac multiorgan function. This study demonstrated a significant association between FALD and III or IV NYHA classes, as well as increased mortality (5.6% vs. 1.3% in non-FALD). Furthermore, we were able to show that conditions promoting poor Fontan circuit hemodynamics are associated with the development of FALD, particularly the absence of fenestration, supraventricular arrhythmias, atrioventricular valve dysfunction, and heart failure [9] [16, 49–51]. Conversely, this analysis did not reveal any associations between the onset of FALD and the type of Fontan operation (*p* = 0.3, adjusted *p*-value = 0.4).

Early-stage Fontan-Associated Liver Disease (FALD) is typically subclinical, prompting universal recommendations for routine liver disease screening, particularly after 10 years from Fontan completion, and earlier if Fontan failure or worsening liver function is observed [10, 38]. In the EUROFontan experience, it is noteworthy that one-third of congenital centers were not fully engaged in liver function screening post-Fontan, indicating that this issue still remains underestimated. Additionally, screening methodologies vary among centers, and no common strategy has been adopted.

Non-invasive liver assessment is a valuable tool for long-term follow-up. In this study, we collected liver data exclusively through non-invasive methods, specifically using ultrasound-based FibroScan. Elastography (either via ultrasound or MRI) evaluates the elastic properties of liver tissue and is widely used to periodically stage hepatic fibrosis and cirrhosis in FALD patients, revealing increased liver stiffness due to varying degrees of fibrosis (52). Since sinusoidal venous congestion can confound ultrasound elastography by overestimating liver stiffness (53), the literature suggests a liver stiffness (LS) measurement of ≥ 22 kPa as a highly effective cut-off predictor for advanced FALD (37). Our study confirms a significant association between FALD and liver stiffness ≥ 22 kPa in transient elastography.

FibroScan-derived liver stiffness measurement is thus a safe and non-invasive diagnostic tool that can be repeated multiple times throughout a patient’s life. Comparing liver stiffness with heart failure severity and hemodynamic parameters to account for any falsely elevated liver stiffness may provide valuable insights for future research.

As previously mentioned, scientific literature suggests a growing prevalence of Fontan-Associated Liver Disease (FALD), especially beyond 10 years post-Fontan surgery, highlighting the need for heightened FALD surveillance after this period. In this series, we show that FALD patients had a longer follow-up duration than non-FALD patients (more than 10 years in 70% of FALD cases versus 53% of non-FALD Fontan cases). Notably, the time elapsed since the Fontan procedure correlates statistically with the degree of collagen deposition and fibrosis, with the risk of Hepatocellular Carcinoma (HCC) increasing exponentially in the third decade [52–54]. International literature reports an HCC prevalence of up to 1.5% in FALD patients [18] with cirrhosis present in only 50% of these cases [29, 55], suggesting that cirrhosis is not an absolute prerequisite for HCC development. There is an annual 1.5–5% risk of HCC in cirrhotic patients [56], with worse prognosis correlating to bridging fibrosis [57] and liver cancer [58–60]. In our study 1.5% of FALD patients died because of a liver-related issue (i.e., liver cancer).

Therefore, it is crucial to determine the best non-invasive and invasive methods for promptly screening and diagnosing cardiac cirrhosis and HCC in FALD patients, establishing its true prevalence, risk factors, and prognostic impact. While liver biopsy is the gold standard for diagnosing adult non-congestive liver diseases, it has several limitations in FALD, including the increased risk of bleeding in congested livers of anticoagulated patients and sampling errors due to the patchy and heterogeneous nature of FALD [52] with ubiquitous liver congestion mimicking features of advanced cirrhosis. Liver biopsy in FALD might be considered as part of follow-up to characterize suspicious liver nodules and prior to heart transplantation (for potential association with liver transplantation) [61]. A precise and universally shared liver surveillance protocol in Fontan patients must be defined in order to depict more comprehensive both invasive and non-invasive liver assessment strategies that can be a jumping-off point for perspective studies on this issue.

In our study FALD was not significantly associated with abnormal liver function tests. However, some international literature reports significant associations with decreased serum albumin and platelet counts, increased levels of gamma-glutamyl transpeptidase, alkaline phosphatase, aspartate aminotransferase, alanine aminotransferase, prothrombin time, indirect bilirubin, and alpha-fetoprotein in HCC cases [10, 25, 29, 50, 62–65]. Other studies indicate that various grades of liver biochemical marker abnormalities exist universally in all Fontan patients (not only in FALD). The clinical significance and appropriate management of these alterations should be supported by laboratory scoring systems and multimodality liver imaging [66].

As demonstrated, severe FALD is associated with a more than threefold increase in mortality [49]. The absence of a shared International Classification of Diseases coded diagnostic and staging protocols may have led to missed FALD diagnoses in this study, affecting the capacity to demonstrate a clear prognostic impact of FALD on survival. Nonetheless, this study confirmed that FALD is significantly associated with late deaths due to liver disease or cancer, emphasizing the urgent need for prospective longitudinal follow-up studies to assess morbidity and mortality risk.

As already stated, the aim of our multicentric European study was to turn the spotlight on FALD as an emerging problem, among the congenital cardiology and cardiac surgery, as well as the gastroenterology communities, problem which is increasing in numbers of patients potentially affected, without being specifically defined in term of diagnosis and work-up. It is therefore essential to develop individualized, multidimensional non-invasive alternatives for the early diagnosis, staging, and monitoring of FALD shortly after Fontan surgery. Consequently, there is a mandatory need for the establishment of multidisciplinary and multicenter research groups to collect the best longitudinal evidence-based data, which will serve as the foundation for developing specific FALD screening/diagnostic criteria, staging systems, and surveillance protocols. The ultimate aim is to define the lifetime risk of clinically significant chronic liver disease to prevent, early diagnose and monitor advanced hepatopathy and hepatocarcinoma, highlighting liver transplantation criteria.

## Study limitations

Although this is the largest international Fontan study focusing on Fontan-Associated Liver Disease (FALD), there are consistent intrinsic limitations due to the retrospective nature of this multicenter study and the lack of commonly worldwide shared specific FALD diagnostic criteria, which have probably introduced an inevitable selection bias. FALD presence was based on questionnaire result (as either “yes/no”) according to aforementioned literature reference suggested by Emamaullee et al. that at the moment provides the more comprehensive FALD diagnostic insights. It makes the data interpretation difficult and it follows that precise instrumental characteristics of liver imaging are unknown, as well as the detailed description of all the specific invasive and non-invasive diagnostic data that allowed for FALD diagnosis for each patient and the exact time of the first FALD diagnosis. All the specific data that supported the diagnosis were not requested in the database collection in view of the extreme variability that may result from the lack of diagnostic criteria. Neverthless the lack of specific diagnostic criteria is a limitation that must emerge in order to provocatively stress the crucial urgent need to worldwide uniform the liver surveillance protocols with the aim to develop perspective studies on this fundamental topic, starting from uniformed surveillance diagnostic-staging protocols in order to minimize selection bias.

Additionally, the data collected span a large historical period and may be affected by varying levels of center-specific FALD awareness, with growing concern in recent decades.

## Conclusions

This analysis, originating from a first European multicenter study, emphasizes the substantial prevalence (16% in this cohort) of Fontan-associated liver disease after Fontan completion, with advanced liver disease/cancer identified as the cause of death in 1.5% of cases. Furthermore, one-third (7 of 21) of the centers are still not fully involved in liver function screening after Fontan, indicating that this issue remains underestimated. The aforementioned limitations reflect the undoubted limitation of both the knowledge and the awareness of the severe liver involvement in the Fontan circulation in a sizeable number of European Centers treating adult patients with congenital heart disease. This study aims therefore to be a starting point for gaining awareness of FALD beginning precisely from the limitations it highlights, which can be a jumping-off point for further essential perspective studies on this issue. This study undoubtedly highlights the urgent need for universally shared diagnostic and staging criteria and mandatory clinical surveillance protocols to promptly diagnose and rigorously monitor FALD throughout a lifelong follow-up. In this setting, liver stiffness measurement appears to be a valuable tool in non-invasive assessment even if it would be interesting to develop perspective studies correlating liver stiffness to hemodynamic parameters.

## Data Availability

No datasets were generated or analysed during the current study.
